# Can the FUT2 *Non-secretor* Phenotype Associated With Gut Microbiota Increase the Children Susceptibility for Type 1 Diabetes? A Mini Review

**DOI:** 10.3389/fnut.2020.606171

**Published:** 2020-12-23

**Authors:** Ottavia Giampaoli, Giorgia Conta, Riccardo Calvani, Alfredo Miccheli

**Affiliations:** ^1^Department of Chemistry, Sapienza University of Rome, Rome, Italy; ^2^NMR-Based Metabolomics Laboratory (NMLab), Sapienza University of Rome, Rome, Italy; ^3^Fondazione Policlinico Universitario 'Agostino Gemelli' IRCCS, Rome, Italy; ^4^Department of Environmental Biology, Sapienza University of Rome, Rome, Italy

**Keywords:** FUT2 gene, T1D, gut microbiota, *secretor*, *non-secretor*, HMOs, bifidobacteria, short-chain fatty acids (SCFA)

## Abstract

The global toll of type 1 diabetes (T1D) has steadily increased over the last decades. It is now widely acknowledged that T1D pathophysiology is more complex than expected. Indeed, a multifaceted interplay between genetic, metabolic, inflammatory and environmental factors exists that leads to heterogeneous clinical manifestations across individuals. Children with *non-secretor* phenotype and those affected by T1D share low abundance of bifidobacteria, low content of short-chain fatty acids, intestinal phosphatase alkaline and a high incidence of inflammatory bowel diseases. In this context, host-gut microbiota dyad may represent a relevant contributor to T1D development and progression due to its crucial role in shaping host immunity and susceptibility to autoimmune conditions. The FUT2 gene is responsible for the composition and functional properties of glycans in mucosal tissues and bodily secretions, including human milk. FUT2 polymorphisms may profoundly influence gut microbiota composition and host susceptibility to viral infections and chronic inflammatory disease. In this minireview, the possible interplay between mothers' phenotype, host FUT2 genetic background and gut microbiota composition will be discussed in perspective of the T1D onset. The study of FUT2-gut microbiota interaction may add a new piece on the puzzling T1D etiology and unveil novel targets of intervention to contrast T1D development and progression. Dietary interventions, including the intake of α-(1, 2)-fucosyl oligosaccharides in formula milk and the use of specific prebiotics and probiotics, could be hypothesized.

## Introduction

Type 1 diabetes (T1D) is a multifactorial autoimmune disease characterized by the loss of pancreatic β-cells that leads to insulin deficiency and hyperglycemia (1). The incidence and prevalence of T1D have risen worldwide over the past 30 years, in particular in children younger than 5 years (1, 2). Several environmental and behavioral factors may have contributed to this trend (e.g., exposure to unhealthy diets, viral and bacterial infections, reduced gut microbiota diversity) (1).

Almost all new T1D diagnoses are characterized by measurable autoantibodies against one or more β-cells protein targets, including insulin, glutamic acid decarboxylase (GAD65), insulinoma-associated protein 2 (IA-2), zinc transporter 8 (ZnT8), and tetraspanin 7 (3, 4). Despite known genetic predisposition, the complex interaction between pancreatic insulin-producing cells and the immune system has not yet clearly established. Moreover, considerable heterogeneity in both symptom presentation and disease progression exist across individuals (1).

Over the last decades, the knowledge of potential T1D etiologic factors has rapidly progressed and novel candidate mechanisms have been identified. Among them, the host-gut microbiota mutual relationship may represent a crucial player in T1D development and progression (5, 6). Indeed, it is now widely recognized that early events associated with the establishment of gut microbiota (e.g., birth mode, breastfeeding, antibiotic use in the first 6 months of age) may shape both the innate and the adaptive immune system (7, 8). Moreover, host-gut microbiota interactions may influence susceptibility to pathogen infections as well as to the development of autoimmune and chronic inflammatory conditions (9).

The fucosyltransferase 2 (FUT2) gene encodes for a critical enzyme responsible for blood group *secretor* status and mucosal protective functions. FUT2 activities prime the composition of glycans on epithelial cell surfaces, on secretory glands and bodily fluids, including the human milk. As a result of the expression of FUT2 gene, four maternal phenotypes have been identified based on the content of fucosylated human milk oligosaccharides (HMOs) (10). The composition of HMOs content and intestinal mucus glycans may profoundly influence gut microbiota dynamics across all life stages and modulate host response to pathogens (10–12).

In addition, FUT2 polymorphisms have been associated with chronic inflammatory conditions and T1D (13). The FUT2 *non-secretor* phenotype was suggested as a factor linking genetic susceptibility to alterations in gut microbiota in T1D etiopathogenesis (Supplementary Material Table 1) (14). Yet, genome-wide association studies (GWASs) did not conclusively support a role of FUT2 gene variants in the crosstalk between the host genotype and gut microbiota in T1D development and progression.

In this mini review, the possible interaction between FUT2 gene variants and gut microbiota in T1D will be discussed. Interventions targeting the FUT2-gut microbiota dyad in T1D are also briefly summarized.

## FUT2 Genetic Variants

The FUT2 gene is located on chromosome 19q13.33 and codes α-(1, 2)-fucosyltranferase enzyme, which catalyzes the transfer of fucose to galactose terminal residues on N-acetylglusamine or N-acetylglucosamine glycan chains. This leads to the expression of H antigen which is the precursor of ABH histo-blood group antigens (HBGAs) in body fluids, including the milk, and in the intestinal mucosa. The synthesis of HBGAs requires several glycosyltranferases acting on precursor oligosaccharides converted into H antigenic structures by fucosylation in α-(1, 2)-linkage. The secretion of H antigen also depends on other glycosyltransferases, such as FUT3 (Le) A/B enzymes, required to synthetize Lewis HBGAs, which are mainly expressed in colon. The FUT2 gene is characterized by several polymorphisms: individuals that are homozygous for the null FUT2 allele do not express ABH antigens in excreta and in the gastrointestinal tract, and are called *non-secretors* or *se*, whereas individuals carrying at least one functional allele are defined *secretors* or *Se*, as they express ABH on secretions (15).

Many polymorphisms in FUT2 show population-specific patterns, yet frequencies of *non-secretor* phenotypes are similar across most populations (16). About 20% of the general population has a *non-secretor* status due to the presence of two inactive variants of the FUT2 gene. Specifically, the single nucleotide polymorphism (SNP) rs601338(A), which codes for a stop codon at position 143 (Trp–Ter), is predominant in Europeans, Iranians, and Africans. On the other hand, the SNP rs1047781(T), which results in the substitution of Ile for Phe at position 129, represents the East Asian counterpart (17). In a Swedish cohort of newborns, the frequencies of selected FUT2 and FUT3 SNPs were assessed and compared with data from the 1000 Genomes Project European Caucasian (EUR) population (18). The frequency of homozygotes for rs601338(A) was 25% and no statistically significant difference was found with the 1,000 Genomes Project cohort (18). Serpa et al. (19) examined FUT2 polymorphisms in a Caucasian population of *non-secretor* individuals from Northern Portugal and evaluated the functional properties of the mutant FUT2 enzymes. Two new polymorphisms in the FUT2 gene (*FUT2-739G*-*A* and *FUT2-839T*-*C*) were identified that lead to the expression of two inactive FUT2 variants (*FUT2-247Gly-Ser* and *FUT2-280Phe-Ser*). A recent study by Soejima and Koda (20) showed that the frequency of putative *non-secretors* was relatively low in Latin American populations except for Puerto Ricans. In the next sections, the role of FUT2 gene variants and associated phenotypes is discussed, with a particular focus on their putative effects on factors associated with T1D development (e.g., viral infections, autoimmune diseases, early life gut microbiota perturbations).

## FUT2 in Pathogen Infections and Autoimmune Diseases

FUT2 gene variants have been associated with numerous conditions, including the susceptibility to bacterial, fungal or viral infections and (chronic) autoimmune diseases, although results are not univocal (21).

Rotavirus (RV) infection is strictly related to host genetics. Indeed, an epidemiological association between the *secretor* status and laboratory-confirmed RV gastroenteritis was observed (22). In various human RV strains, analyses of the carbohydrate binding properties of VP8^*^ (the spike protein domain that mediates viral attachment) revealed a specific affinity for neutral oligosaccharides of the HBGAs (23). *In vitro* studies, using various glycan binding assays, have demonstrated that the most common human RVs recognize HBGAs through VP8^*^ (24).

Regarding bacterial infections, links between FUT2 *secretor* status and *Helicobacter pylori (H. pylori)* ability to attach to human gastric mucosa were found. Mucosal glycans carrying blood group A, B or H antigens are known to be ligands for *H. pylori* and *secretor* individuals may be more susceptible to *H. pylori* infection (25). Recent findings by Rossez et al. (26) confirmed that gastric mucins from *secretor* people interact more efficiently with *H. Pylori* than those from *non-secretors*.

Noroviruses (NoVs) are the major causative agents of acute non-bacterial gastro-enteritis (27). Interestingly, some individuals do not develop infection, despite being exposed to high viral loads (28). A study performed on 77 volunteers showed that HBGAs were critical factors for NoV infection (27). In particular, the presence of inactive FUT2 alleles was responsible for this innate genetic resistance (27). Furthermore, the *non-secretor* phenotype has been associated to a slow progression of human immunodeficiency virus 1 (HIV-1) infection (29). Indeed, the lack of fucosylation may prevent the formation of glycolipid receptors for HIV-1 on the epithelial surface (29). Conversely, the *non-secretor* phenotype may confer higher susceptibility to *Candida albicans* (30), *Streptococcus Pneumoniae* (31), and mumps infection (32).

In addition, the *non-secretor* status was associated with self-reported kidney disease (32) and higher risk of celiac disease (33).

Autoimmune diseases are particularly burdensome due to their health, social and economic consequences (34). The FUT2 *non-secretor* phenotype has been associated with complex and multifactorial disorders, such as Behçet's disease, and inflammatory bowel diseases (IBDs), including Crohn's disease (CD) and ulcerative colitis (UC) (35, 36). CD is a chronic, relapsing inflammatory disease, mainly affecting the small intestine and colon. CD pathogenesis involves multiple interacting elements, such as genetic susceptibility factors, priming by the enteric microflora, and immune-mediated tissue injury (37). Results of a study by McGovern et al. (38) provided evidence that *non-secretor* status increased CD susceptibility in the European population. A study carried out on Caucasian individuals has shown that *non-secretors* are at high risk of developing CD, albeit no significant association between ABO variants and CD was detected (39).

Urine profiling analyses conducted by Rueedi et al. (40) on 835 individuals showed that urinary fucose was associated with a FUT2 gene variant linked to CD. Furthermore, elevated urinary concentrations of fucose, also observed in *non-secretors*, may reflect changes in gut flora activities shifting from a healthy status toward CD. Urinary fucose may thus represent an early biomarker of CD.

However, robust evidence linking FUT2 gene variants to autoimmune diseases is still lacking. A recent study on 635 people with CD did not find an association between rs601338(A) and disease phenotype, severity, or clinical outcomes, suggesting that lack of fucosylation may be involved in CD development rather than in disease progression (41). In contrast, in people with UC, functional FUT2 variants seem to contribute to the different clinical manifestations of the disease (42).

Although the current knowledge does not support a clear-cut association between FUT2 gene variants and the development and progression of autoimmune diseases, the interaction between FUT2 phenotype and the immune system is a promising target to be explored.

## FUT2, Gut Mucus Glycans and Human Milk Oligosaccharides in Health and Disease

Genetics contribute to determining the chemical composition of the intestinal mucus layer, which, in turn, modulates gut microbiota composition and host innate immunity (43). In this context, a number of studies assessed whether FUT2 gene variants may influence the quality and quantity of microbiota in infants with conflicting results. The *secretor* status was associated with higher diversity, richness and abundance of gut *Bifidobacterium* and *Bacteroides* compared to *non-secretor* individuals during the first months of life (44–47). In both people with loss-of-function alleles of FUT2 gene and FUT2 null mice, specific microbiome, meta-proteome and meta-metabolome signatures were described (48). In particular, the gut microbiome in *non-secretors* was characterized by a depletion in amino acid metabolism pathways and an overall enrichment in genes encoding for carbohydrate and lipid metabolism, glycan biosynthesis, and catabolism (48). These features were paralleled by changes in metabolic profiles. Interestingly, the microbial compositional and functional signature associated with *non-secretor* phenotype was associated with sub-clinical intestinal inflammation (48).

However, the actual role of FUT2 gene variants in shaping the activity and composition of the gut microbiome is still disputed. Indeed, recent large observational studies as well as GWASs found no or weak association between FUT2 genotype and stool microbiome composition (49–52). In a large cohort of 1,190 healthy young adults, FUT2 genotype and *secretor* status were not associated with fecal microbial alpha diversity, composition or inferred microbial function (49). In 1,503 individuals from a twin cohort in the United Kingdom, a consistent link between the taxonomic composition of microbiota and the *secretor* status was not evident (50). Furthermore, in a large cohort from the Genetic Environmental Microbial (GEM) Project, GWAS of 3,727,707 SNPs found 58 SNPs associated with the relative abundance of specific microbial taxa (51). FUT2 gene variants were not present among the SNPs that showed an association with the gut microbiome (51). Finally, in fecal samples from two independent German cohorts, weak associations were found for FUT2 with *Clostridium* IV and unclassified *Clostridiales* (52), in partial agreement with Wacklin et al. (44). However, the contribution of FUT2 gene variants in terms of overall influence on microbial variation was negligible (52).

Maternal FUT2 gene may also play a role in early phases of the colonization of the infant gut through its action on the composition of HMOs (53, 54). HMOs represent the third most abundant component of the breast milk. HMOs are minimally digested by the host, and are believed to influence biochemical processes in the infant's gut (10). The expression of the FUT2 gene, codingα-1,2-fucosyltransferase, and FUT3 gene, coding α-1,3/1,4-fucosyltransferase, affects directly HMOs content. Fucosylated oligosaccharydes such as 2′-fucosyllactose (2′ FL), 3′-fucosyllactose (3′ FL), and lacto-N-fucopentaose I/II/III (LNFP I/II/III) are higher in *secretors* compared to *non-secretors* (12, 55, 56) but, in general, the HMOs content changes during the lactation period (57). Chemical structures of HMOs are strictly related to their prebiotic effects and their putative role in inhibiting pathogen infections (58, 59). Free HMOs can mimic host receptors and bind directly to pathogens, thus allowing their elimination through feces. HMOs can also compete with pathogens for the binding to the host cell-surface glycan receptors (11, 12, 57).

**Figure 1:**
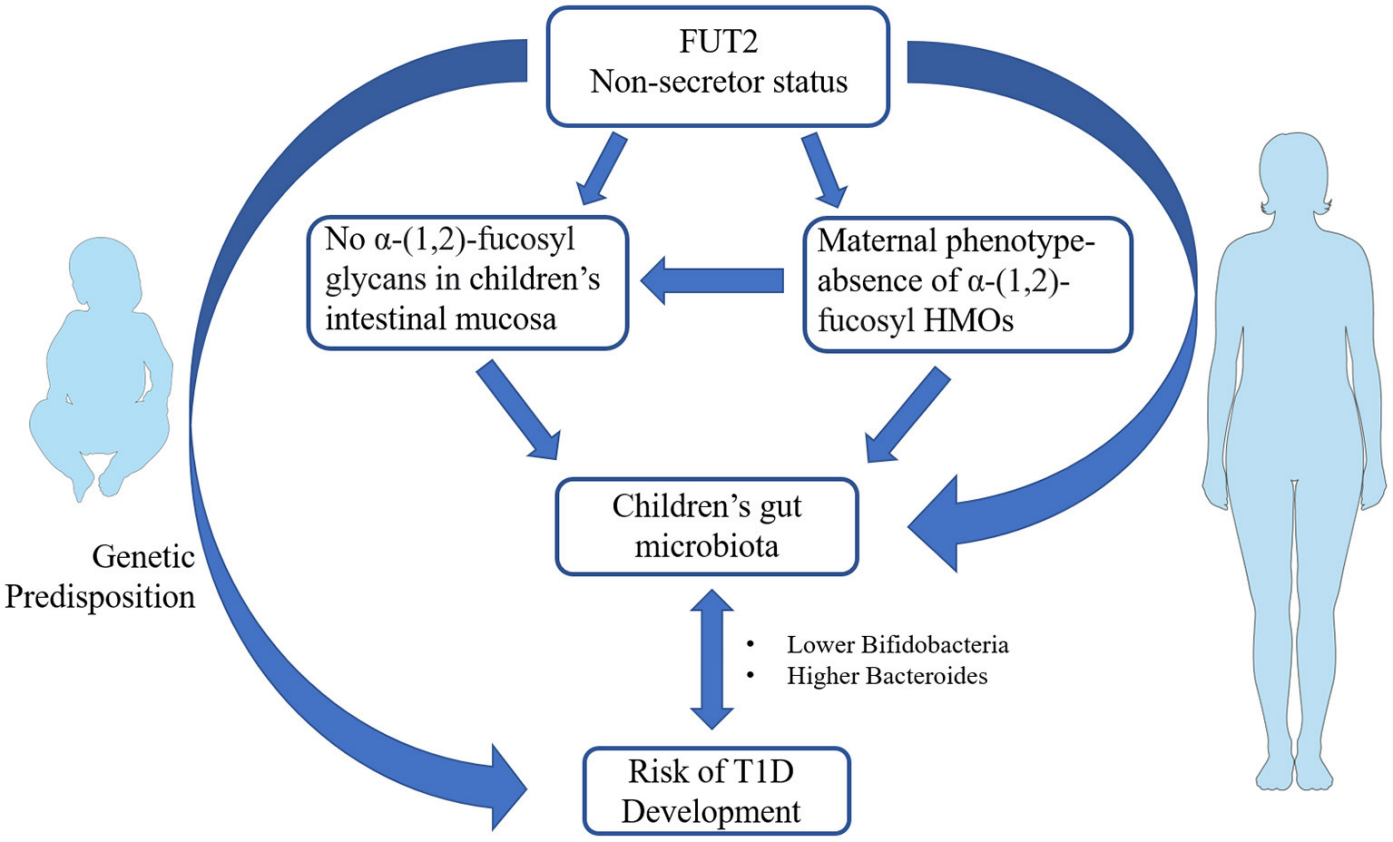
Schematic representation of the potential link between fucosyltransferase 2 (FUT2) *non-secretor* phenotype and infants' gut microbiota in the pathogenesis of type 1 diabetes (T1D). FUT2 genetic variants coding for *non-secretor* status has been associated with increased T1D susceptibility. *Non-secretor* status in mothers influence the composition of human milk oligosaccharides (HMOs) that in turn may prime the composition of newborns' gut microbiota. The occurrence of *non-secretor* status in infants modulates the glycan composition of intestinal mucosa and therein gut microbiota establishment. *Non-secretor* children and/or children breastfed by *non-secretor* mothers show a lower number of bifidobacteria and a higher content of *Bacteroides* in their gut. The same gut microbiota profiles are present in children with T1D. The figures were drawn using the freely available Servier Medical Art Resource (https://smart.servier.com/).

Previous works showed that infants breastfed by *secretor* women exhibited a higher number of bifidobacteria, which commonly use HMOs as a carbon source, and in particular 2′ FL, 3′ FL and lactodifucotetraose (LDFT) (60). Some studies reported beneficial effects related to the intake of HMOs. For instance, 2′FL enhanced brain function, learning and memory in rats (61) and protected mice against necrotizing enterocolitis (NEC) (62). 2′ FL may also protect infants against *Campylobacter*-induced diarrhea (63, 64), while lacto-N-difucohexaose I (LNDFHI) has a positive effect in mitigating NV-related diarrhea (65). Milk from *non-secretor* mothers lacks 2′ FL and related fucosyl oligosaccharydes, and has been shown to exert a lower protection against enteropathogens that bind α-(1, 2)-fucose (66). A lower content in bifidobacteria taxa has been determined in gut microbiota of infant breastfed by *non-secretor* mothers. Intriguingly, a similar gut microbiota composition is also present in children with T1D (67), suggesting that a FUT2-dependent priming of gut microbiota may also be present in T1D (Figure 1). Several studies on newborn nutrition demonstrated that breastfeeding from *secretor* mothers is the most efficient way to transfer fucosylated HMOs to infants (33, 52, 53). Hence, investigations were conducted to evaluate if the addition of specific α-(1, 2)-fucosylated HMOs to formula milk may have the same beneficial effects on gut microbiota. Studies on specific HMOs supplementation in formula milk showed that 2′ FL resulted safe for infants up to 1 year of age (68) and led to a reduction in inflammatory cytokines (69). Yet, evidence correlating α-(1, 2)-fucosylated HMOs in formula milk and gut microbiota in the context of autoimmune diseases are presently lacking. Genetics contribute to determining the chemical composition of intestinal mucus layer, which, in turn, modulates gut microbiota composition and host innate immunity (70).

Through the modulation of infant mucosal glycans, HMOs may also affect intestinal cell maturation and barrier function (71). An increased intestinal permeability allows pathogens, toxins or food antigens to translocate from the intestinal lumen to extraintestinal compartments leading to intestinal disorders, allergies and diseases including T1D, IBD, irritable bowel syndrome, and metabolic syndrome (72, 73). HMOs produced by *secretor* mothers may stimulate the growth of specific bifidobacteria that in turn release peptides able to enhance the expression of tight-junction protein (74). Other milk-derived peptides have shown beneficial effects on intestinal barrier function, supporting the crucial role of lactation in promoting intestinal mucosal homeostasis (75).

Future investigations are needed to clearly establish the role of FUT2 gene variants on gut microbiota composition. Moreover, further studies should determine if HMOs bifidogenic properties may be exploited by *non-secretor* mothers or mothers who are unable to breastfeed to promote a healthier gut environment in newborns.

## FUT2, T1D and Microbial Composition and Metabolites

The multifactorial etiopathogenesis of T1D poses a major challenge for the implementation of personalized therapeutic strategies (76). As for T1D genetics, several susceptibility genes have been identified that vary across genders, racial groups, and geographical regions (1). Main genetic risk factors are located within the class II HLA region (77). In a meta-analysis of GWASs combining British and American cases and controls, FUT2 was not present among newly identified T1D loci (78). Additional T1D susceptibility genes were identified by researchers from the Type 1 Diabetes Genetics Consortium through the application of an extensive GWAS on larger sample of T1D cases (79, 80). Most candidate loci that passed the stringent genome-wide significance levels pertained to the inflammatory cytokine network (79, 80). Interestingly, the ABO blood group locus, that may influence the glycosylation patterns of cells at gastrointestinal mucosal lining, was associated with positivity to antibodies against gastric parietal cells (PCA) in T1D patients (80). However, in the same study, when FUT2 SNP rs601338(A) was tested for a potential association with PCA only unconvincing evidence was showed (80).

Although those GWASs and sequencing studies did not include FUT2 out of over 60 loci associated with T1D susceptibility, FUT2 SNPs have been retrieved among the numerous gene polymorphisms outside HLA regions associated with T1D risk (76). Smyth et al. (13) provided evidence of a recessive association between FUT2 SNP rs601338(A) and T1D in European populations. Further association between FUT2 gene variants and T1D was found in the study conducted by Ihara et al. (81), that examined the contribution of the FUT2 gene and ABO blood type in Japanese children. The SNP rs1047781(T) coding for *non-secretor* phenotype was found to confer susceptibility to T1D (81). Intriguingly, children with HLA-conferred disease susceptibility that are homozygous for the functional FUT2 allele G (coding for *secretor* phenotype) are predisposed to rapid disease progression (82).

Both genetic and environmental factors are involved in T1D pathogenesis (76). In particular, host genetics and gut microbiota may affect T1D development through their influence on metabolic and immune phenotypes (51, 83). Gut microbiota and bacterial-derived components are critical to educate immune system function after birth (84) and may be involved in the progression from β-cell autoimmunity to overt T1D (85). In non-obese diabetic (NOD) mice, specific gut microbial features and products influence the pancreatic immune environment and confer protection against T1D (86–88). In humans, T1D status has been associated with an increase in *Bacteroides* and *Bacteroidetes*-to-*Firmicutes* ratio (67, 89–91), and with a dearth of *Bifidobacterium* species (67, 90). In particular, a reduction in butyrate-producing species was found in children with β-cell autoimmunity (92). In The Environmental Determinants of Diabetes in the Young (TEDDY) study, the gut microbiome of children with islet autoimmunity and T1D showed fewer genes related to short-chain fatty acids (SCFA) biosynthesis than that of healthy controls, although taxonomic differences were modest and not significant (6). Bacterial-derived SCFA, such as acetate, propionate and butyrate, which originate from the microbial fermentation of several dietary substrates, including HMOs. SCFA are then absorbed at the colon epithelium level and influence both local and systemic metabolic and immune responses, as well as improving the intestinal barrier functionality by facilitating tight junction assembly (93, 94). Lactic acid bacteria belonging to *Bifidobacterium* genus are capable of breaking HMOs and are transferred from mother to child during delivery. In particular B. infantis are efficient at metabolizing HMOs into SCFA. Interestingly, a reduced abundance of lactate-producing bacteria has been observed in T1D patients (95).

In NOD mice, a 6-week supplementation with a mixture of HMOs in early life protected against T1D development and halted severe pancreatic insulitis later in life (89). HMOs effects were associated with beneficial changes in fecal microbiota composition and activity (e.g., high content of SCFA in stool and cecum) and reduction in pro-inflammatory diabetogenic cytokines (89). Moreover, HMOs combined with SCFA may modulate dendritic cells and prime functional regulatory T cells *in vitro* toward a tolerogenic phenotype (89).

Collectively, these findings suggest that perturbations in the gut microbial function may alter host-microbial metabolism and lead to the development of a “leaky” gut followed by autoimmunity (96). HMOs in human milk may confer protection to children at risk for T1D by priming immune and gut microbiota development in early life.

Timely interventions on gut microbiota may represent a promising strategy to reduce the risk of T1D (97). However, data on the effects of probiotics administration in children with T1D are scarce. In a multinational cohort study of children at increased genetic risk of T1D, reduced pancreatic islet autoimmunity (PIA) was observed in those who received probiotics before the first month of age (98). Early probiotic administration was associated with a 60% reduction in PIA risk in children at the highest genetic risk of T1D (98).

Further studies are needed to examine the association between *Bacteroides* and bifidobacterial species in *non-secretors* and T1D to identify specific prebiotic and probiotic mixtures with preventive and therapeutic function.

Several metabolites derived by host-microbiome interaction may modulate immune response to pathogens through the activation of numerous receptors in the intestinal epithelial cells (99).

In this context, significant changes in gut microbial metabolic pathways have been found in children with T1D. An increase in the multiple sugar transport system, which is involved in the utilization of d-galactose, d-xylose, l-arabinose, d-glucose, and d-mannose, and a decrease in the biosynthesis of a number of amino acids, in particular Tyr and Phe, have been observed (100). Furthermore, serum metabolic profiling studies in individuals who developed T1D showed reduced levels of succinic acid, phosphatidylcholine and ketoleucine and increased levels of glutamic acid months before autoantibody positivity. Autoimmunity may thus represent a later manifestation of early-life alterations, including metabolic perturbations (101).

In a recent study, people with T1D and IBD were found to exhibit shared intestinal inflammatory patterns. Individuals with T1D showed higher fecal calprotectin levels and lower fecal intestinal alkaline phosphatase (IAP) activities associated with lower levels of butyrate and propionate, as compared with non-diabetic controls (102). It is known that IAP plays an important role in the interactions among diet, gut microbiota composition, and inflammatory responses in the gastrointestinal tract (103). IAP regulates lipid transport through intestinal mucosa and interferes with bacterial translocation across the intestinal epithelium, thereby preventing the consequent inflammatory response. The expression and secretion of IAP also depend on diet and intestinal microbiota (103). Serum IAP activity was found to be interconnected with ABO blood groups and FUT2 *secretor*/*non-secretor* phenotypes (104). People with the FUT2 *secretor* phenotype also tend to have higher serum IAP activity levels after a meal compared with *non-secretors* (105).

In conclusion, host genetic factors, gut microbiota activities, and host-gut co-metabolism may all contribute to the development of β-cell autoimmunity and T1D. FUT2 may represent a plausible player in this complex puzzle. Yet, conflicting results exist on the relationship between FUT2-associated *secretor* phenotype and gut microbiota composition and function. Further studies are needed to clearly establish whether FUT2 variants shape the gut microbiome and affect T1D development and progression.

## Conclusions

The interplay between FUT2 variants and associated glycan profiles, gut microbiota composition and function, and host inflammatory response may play a role in T1D pathogenesis. Recent studies have spurred interest on the subject for its possible clinical implications (14). FUT2-associated phenotypes may be associated with T1D pathogenesis at different levels and timeframes and this may offer new possibilities for both primary prevention started early in infancy and secondary prevention when islet autoimmunity has ensued. Maternal FUT2 status should be assessed and beneficial bifidogenic properties of supplemented HMOs formula may be exploited in case of *non-secretor* phenotypes. A comprehensive and conclusive analysis on the effects of FUT2 gene variants on children gut microbiota composition, inflammatory status and β-cell autoimmunity may provide relevant information for T1D disease risk stratification and early interventions (e.g., tailored diets, specific prebiotics, probiotics or symbiotic) to preserve gut integrity and impede autoimmune response. Gut microbiota of T1D children with FUT2 susceptibility SNPs could be targeted through the supplementation with α-(1, 2)-fucosyl-oligosaccharides enriched formula milk or specific prebiotics and probiotics to stimulate the intestinal production of SCFA or IAP.

However, conclusive evidence relating FUT2 status and T1D has yet to be achieved and more research is required in this area.

## Author Contributions

OG, GC, RC, and AM designed the study, made the description plan, performed manuscript writing and figure charting, and actively participated to the discussions. RC and AM made arrangements on the manuscript according their discussions and reviewed critically this article. All authors contributed to the article and approved the submitted version.

## Conflict of Interest

The authors declare that the research was conducted in the absence of any commercial or financial relationships that could be construed as a potential conflict of interest.
