# Supplementary material for: Can the FUT2 Non-secretor Phenotype Associated With Gut Microbiota Increase the Children Susceptibility for Type 1 Diabetes? A Mini Review
Source: Front Nutr. 2020 Dec 23;7:606171. doi: 10.3389/fnut.2020.606171 (PMC7785815; doi:10.3389/fnut.2020.606171)
Supplement: Supplementary file 1 [file Data_Sheet_1.PDF]

Supplementary Material

Supplementary Table 1. Main research studies reporting the association between fucosyltransferase 2 (FUT2) and autoimmune diseases, gut microbiota composition and type 1 diabetes (T1D).

Study population	Study type	Main findings	Reference
**FUT2 and autoimmune diseases**			
6,333 individuals affected by Crohn's disease and 15,056 controls from Europe and USA	GWAS	FUT2 was identified as a genome-wide significant risk locus for Crohn's disease	Franke A.[35]
292 Iranian Behçet's disease (BD) cases and 294 age- and sex-matched controls	GWAS	FUT2 gene variants were associated with BD in independent Iranian and Turkish datasets	Xavier J.M.[36]
635 patients with Crohn's Disease	Longitudinal cohort study	FUT2 genotype was not associated with disease phenotype, disease severity, or clinical outcomes.	Wang W.[41]
**FUT2 and T1D**			
8,344 patients with T1D and 10,008 control subjects from UK, and 3,360 type 1 diabetic families from Europe, North American and Asian-Pacific regions.	Case-control study	The *non-secretor* A/A genotype (rs601338) confer susceptibility to T1D in both the case-control and family collections (odds ratio for AA 1.29 [95% CI 1.20–1.37] and relative risk for AA 1.22 [95% CI = 1.12–1.32].	Smyth D.J.[13]
305,090 SNPs tested in 3,561 T1D cases and 4,646 controls from US and UK cohorts	Meta-Analysis of three GWAS	Fifteen T1D risk loci identified. FUT2 was not present among candidate susceptibility gene	Cooper J.D.[78]
7,514 T1D cases and 9,045 control subjects	GWAS and meta-analysis	Over 41 loci associated with T1D were identified, including IL10, IL19, IL20, GLIS3, CD69 and IL27. FUT2 gene variants were not included among susceptibility genes	Barrett J.C.[79]

8,506 people with T1D as part of the Juvenile Diabetes Research	GWAS	Two loci showed remarkable association with islet autoimmunity: the FCRL3 locus and the ABO blood group locus	Plagnol V.[80]
531 Japanese children with T1D. 448 Japanese adults without diabetes and 1216 individuals in a general Japanese population.	Case-control study	The *non-secretor* se2 genotype (c.385A>T) of the *FUT2* gene was found to confer susceptibility to Type 1A diabetes in a recessive effects model [odds ratio for se2/se2, 1.68 (95% CI 1.20–2.35); corrected *P* value = 0.0075].	Ihara K.[81]
7410 HLA-predisposed children participating in the Finnish Type 1 Diabetes Prediction and Prevention (DIPP) study	Observational Study	Individuals with rapid progression to T1D have a higher prevalence of the *secretor* genotype in the FUT2 gene among children carrying the high-risk HLA genotype	Pöllänen P.M.[82]
41 nondiabetic controls and 46 patients with T1D from the FinnDiane Study.	Case-control study	The distribution of ABO blood antigen groups or FUT2 *secretor/non-secretor* phenotypes did not differ between T1D and controls.	Lassenius M.I.[102]
**FUT2 and gut microbiota**			
Eighty-two healthy adult individuals from Helsinki metropolitan area, Finland.	Observational study	The *non-secretor* individuals showed lower amount of *B. bifidum*, *B. adolescentis* and *B. catenulatum/pseudocatenulatum* species.	Wacklin P.[44]
Twenty-five healthy Spanish mothers with exclusive breast-feeding practices	Pilot study	The predominant HMOs in *secretor* milk samples were 2’FL and lacto-N-fucopentaose I, whereas *non-secretor* milk was characterized by lacto-N-fucopentaose II and lacto-N-difucohexaose II. *Lactobacillus* spp, *Enterococcus* spp, and *Streptococcus* spp were lower in non-*secretor* than in *secretor* samples. Beneficial *Bifidobacterium* genus and species were less prevalent in *non-secretor* samples.	Cabrera-Rubio R.[45]
Feces samples from two full-term infants from U.S.	Proof-of-concept study	FUT2 *secretor* status of mothers influence the composition of HMOs that in turn shape the newborns’ gut microbiota population. FUT2 *secretor* status has a beneficial bifidogenic activity.	De Leoz M.L.A.[57]
A total of 99 children (47 Indians, 52 Finnish) of 13-14 years of age.	Cross-sectional comparative study	FUT2 *secretor* status did influence the gut microbial composition in both the cohorts; *Bifidobacteriaceae* and *Verrucomicrobiacea e* were significantly abundant in *secretors* as compared to *non-secretors*. bacterial families like *Coriobacteriaceae,*	Kumbhare S.V.[47]

		Prevotellaceae and *Clostridiaceae* were highly abundant in *secretors* when compared to *non-secretors* of the Indian population, while *Lachnospiraceae* group was highly significant in *secretor* children within Finnish cohort.	
Stool samples from 1,190 healthy individuals of European descent from Canada, Israel and USA	Observational study	FUT2 genotype and secretor status are not associated with human fecal microbial composition.	Turpin W.[49]
1503 individuals from a cohort of twins from the United Kingdom	Observational study	Taxonomic composition of the microbiota was not associated with secretor status.	Davenport E.R.[50]
1,561 subjects from the Genetic Environmental Microbial (GEM) Project	Observational Study	Fifty-eight SNPs were associated with the relative abundance of 33 taxa. FUT2 gene variants were not associated with intestinal microbial composition.	Turpin W.[51]
Two independent German cohorts of 914 individuals (PopGen) and 1,115 individuals (Food-Chain Plus; FoCus)	GWAS	Several genetic and non-genetic factors associated with the composition of the human gut microbiome were identified. FUT2 gene variants were associated with intestinal taxonomic classification.	Wang J.[52]
Thirty-three stool samples from healthy subjects recruited from Rush University Medical Center (RUMC), Chicago, Illinois.	Observational study	*Secretor* status may shape gut bacterial composition in health. Lower abundances of Lachnospiraceae family noted in *non-secretors* compared with *secretors.* Lower genus *Blautia* and higher abundances of members of the Rikenellaceae, Peptostreptococcaceae, Clostridiales, and *Turicibacter* were found in *secretors.*	Gampa A.[70]
**T1D and gut microbiota**			
783 children followed from three months to up to 5 years old, from six clinical centers in four countries (Finland, Germany, Sweden and the United States) who either progressed to persistent IA or T1D or were matched as controls.	Prospective cohort case-control study	In T1D case–control comparisons, controls had higher levels of *Streptococcus thermophilus* ($q = 0.078$) and *Lactococcus lactis* ($q = 0.094$) species, whereas cases contained higher levels of species such as *Bifidobacterium pseudocatenulatum* ($q = 0.078$), *Roseburia hominis* ($q = 0.11$) and *Alistipes shahii* ($q = 0.14$), supporting the protective effects of SCFAs in early-onset human T1D.	Vatanen T.[6]

12,500 stool samples from 903 children with IA, T1D and matched controls from three European countries (Germany, Sweden and Finland) and three US states (Colorado, Georgia and Washington).	Case-control study	In the T1D and control cohort, five bacterial genera were associated with T1D onset, with *Parabacteroides* the most significant ($P < 0.001$). Eleven bacterial genera were lower in T1D cases, including four unclassified Ruminococcaceae, *Lactococcus* ($P = 0.020$), *Streptococcus* ($P = 0.032$), and *Akkermansia* ($P = 0.045$) .	Stewart C.J.[8]
Eighteen children with HLA-conferred susceptibility to T1D and 18 controls matched for age, sex, and HLA-DQB1 genotype.	Case-control study	A low abundance of lactate-producing and butyrate-producing species was associated with β-cell autoimmunity. Low *Bifidobacterium adolescentis* and *Bifidobacterium pseudocatenulatum*, and increased *Bacteroides* genus were observed in the children with β-cell autoimmunity.	de Goffau M.C.[67]
Stools from 8 Finnish children at different points (between 3 and 40 months of age).	Case-control study	Children who developed autoimmunity has a microbiome that is less diverse and stable. *Bacteroides ovatus* accounts for 24% of the total increase in the phylum Bacteroidetes in cases compared with controls.	Giongo A.[89]
Sixteen Caucasian children with T1D and sixteen matched controls.	Case-control study	In children with T1D, the bacterial number of Actinobacteria and Firmicutes, and the Firmicutes to Bacteroidetes ratio were significantly decreased and related to the glycemic level. Gut microbiota changes associated with reduced gut integrity in children with T1D.	Murri M.[90]
Twenty-eight children with T1D and 27 age-matched control from France, Greece, Estonia, and Finland.	Case-control study	Non-diabetic children have a more balanced microbiota with higher abundance of butyrate-producing species than children with T1D.	de Goffau M.C.[91]
7473 children from the TEDDY study.	Prospective cohort study	Early probiotic supplementation was associated with 60% decrease in the risk of IA in children at the highest genetic risk of T1D.	Uusitalo U.[98]
Thirty-three stool samples and serum samples collected from genetically predisposed T1D children, recruited to the study cohort from Espoo, Finland (n = 27) and Tartu, Estonia (n = 6).	Observational study	Low gut microbiota diversity was observed in T1D subjects before the occurrence of clinical manifestations. T1D-associated microbiota promoted a metabolic environment that lead to inflammation and disease progression.	Kostic A.D.[100]
